# Interleukins and their signaling pathways in the Reactome biological pathway database

**DOI:** 10.1016/j.jaci.2017.12.992

**Published:** 2018-02-21

**Authors:** Steve Jupe, Keith Ray, Corina Duenas Roca, Thawfeek Varusai, Veronica Shamovsky, Lincoln Stein, Peter D’Eustachio, Henning Hermjakob

**Affiliations:** a European Molecular Biology Laboratory, European Bioinformatics Institute (EMBL-EBI), Wellcome Genome Campus, Hinxton, United Kingdom; b VHsquared, Cambridge, United Kingdom; c NYULangone Medical Center, NewYork, NY; d Ontario Institute for Cancer Research, Toronto, and the Department of Molecular Genetics, University of Toronto, Toronto, Ontario, Canada; e State Key Laboratory of Proteomics, Beijing Proteome Research Center, National Center for Protein Sciences-Beijing (PHOENIX Center), Beijing Institute of Radiation Medicine, Beijing, China

**Keywords:** Interleukins, pathways, signaling, database, Reactome, diagram, illustration

## Abstract

**Background:**

There is a wealth of biological pathway information available in the scientific literature, but it is spread across many thousands of publications. Alongside publications that contain definitive experimental discoveries are many others that have been dismissed as spurious, found to be irreproducible, or are contradicted by later results and consequently now considered controversial. Many descriptions and images of pathways are incomplete stylized representations that assume the reader is an expert and familiar with the established details of the process, which are consequently not fully explained. Pathway representations in publications frequently do not represent a complete, detailed, and unambiguous description of the molecules involved; their precise posttranslational state; or a full account of the molecular events they undergo while participating in a process. Although this might be sufficient to be interpreted by an expert reader, the lack of detail makes such pathways less useful and difficult to understand for anyone unfamiliar with the area and of limited use as the basis for computational models.

**Objective:**

Reactome was established as a freely accessible knowledge base of human biological pathways. It is manually populated with interconnected molecular events that fully detail the molecular participants linked to published experimental data and background material by using a formal and open data structure that facilitates computational reuse. These data are accessible on aWeb site in the form of pathway diagrams that have descriptive summaries and annotations and as downloadable data sets in several formats that can be reused with other computational tools. The entire database and all supporting software can be downloaded and reused under a Creative Commons license.

**Methods:**

Pathways are authored by expert biologists who work with Reactome curators and editorial staff to represent the consensus in the field. Pathways are represented as interactive diagrams that include asmuchmolecular detail as possible and are linked to literature citations that contain supporting experimental details. All newly created events undergo a peer-review process before they are added to the database and made available on the associatedWeb site. New content is added quarterly.

**Results:**

The 63rd release of Reactome in December 2017 contains 10,996 human proteins participating in 11,426 events in 2,179 pathways. In addition, analytic tools allow data set submission for the identification and visualization of pathway enrichment and representation of expression profiles as an overlay on Reactome pathways. Protein-protein and compound-protein interactions from several sources, including custom user data sets, can be added to extend pathways. Pathway diagrams and analytic result displays can be downloaded as editable images, human-readable reports, and files in several standard formats that are suitable for computational reuse. Reactome content is available programmatically through a REpresentational State Transfer (REST)-based content service and as a Neo4J graph database. Signaling pathways for IL-1 to IL-38 are hierarchically classified within the pathway “signaling by interleukins.” The classification used is largely derived from Akdis et al.

**Conclusion:**

The addition to Reactome of a complete set of the known human interleukins, their receptors, and established signaling pathways linked to annotations of relevant aspects of immune function provides a significant computationally accessible resource of information about this important family. This information can be extended easily as new discoveries become accepted as the consensus in the field. A key aim for the future is to increase coverage of gene expression changes induced by interleukin signaling

## Graphical abstract

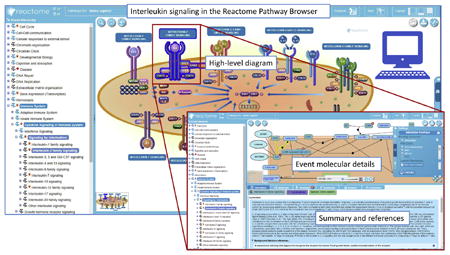

At the cellular level, life is a network of molecular events that includes processes, such as signal transduction, transport, and metabolism. Reactome represents these processes as pathways, connected events termed reactions that define changes in the state of biological molecules. Reactions include binding; translocation; posttranslational modifications, such as phosphorylation; and biochemical reactions. The molecular events defined by reactions connect to represent ordered transformation networks. Reactome represents human biological events in graphic pathway diagrams supported by descriptions, references, and links to external resources. The result is an extension of the concept underlying classic metabolic maps, diagrams that represent a broad range of physiologic processes underpinned by data that are freely available in a formally defined and consistent structure that is computationally reusable.^1^

By placing human proteins in pathways, Reactome characterizes their molecular functions, providing a resource that is both an archive of biological processes and a tool for discovering functional relationships within user data sets. Reactome pathways include (December 2017, version 63) 10,996 human proteins. Reactome pathway descriptions, images, and underlying data can be downloaded easily in a variety of human-readable and computationally reusable formats. In addition to the Web site, Reactome provides programmatic access tools through content and analysis services.

Reactome is a free database of human biological pathways available through the Reactome Web site (https://reactome.org), where content can be searched, viewed, and downloaded. Pathways are annotated manually by professional curators and peer reviewed by expert biologists. The aim is to represent the consensus view for each area of biology in a reliable and consistentmanner such that the underlying data have a structure amenable to use both as a reference knowledge base and as a computational resource for high-throughput querying and reuse. Pathways are represented in as much mechanistic detail as possible, detailing every established molecular event. Events are termed reactions but are not limited to classical biochemical events; binding, dissociation, and translocation between cellular compartments and all forms of posttranslational modification, such as phosphorylation, are also considered reactions.

On the Reactome Web site, pathways are organized hierarchically, following, where possible, the Gene Ontology Biological Process classification. The uppermost levels of the hierarchy represent broad topics in biology, such as the immune system, and serve to group together as subpathways the narrower topics contained within them. At lower hierarchical levels, the pathway topics become more focused and are represented in full molecular detail in interactive pathway diagrams. All pathways are represented as graphics with associated descriptions, literature references, and links to further annotation sources covering areas such as expression, structure, genetics, and disease. Reactome also provides analytic tools that can be used to submit a user data set for overrepresentation analysis, visualize expression data as an overlay on Reactome pathway graphics, or compare Reactome’s human pathways with predicted pathways in model organisms.

All of Reactome’s content, including graphics and results of analyses, can be downloaded in editable formats for reuse in reports and publications, and the images of molecules and cellular components are available as an icon library for use and extension by the community.

Reactome’s coverage of interleukins, their receptors, and signaling pathways has recently been extended to cover IL-1 to IL-38. The classification used is largely derived from Akdis et al.^2^ Human interleukins, which were first identified as mediators of signaling between leukocytes (white blood cells),^3^ are a family of secreted proteins that function as ligands for receptors that mediate signaling among cells of the immune system.^2^ Certain interleukins have chemokine- or interferon-like properties, but their most important role is as mediators of the physiologic response to infection. Interleukins initiate signaling by binding to receptors located on the cell surface. Their function is as a local signal (paracrine or autocrine) rather than as a circulatory long-range (endocrine) signal, which differentiates them from the steroids and peptide hormones. The response of a cell depends on the specific interleukin involved, the receptors it expresses on its surface, and the signaling cascades that become activated. This physiologic role, as well as their mechanism of action as ligands that bind to cell-surface receptors, makes them attractive drug targets, and they are the subject of much active pharmaceutical and clinical research.

Interleukins have been classified into families based on a variety of characteristics, including biological function, shared receptor components, sequence homology, structural motifs, and cellular origin. Alternative nomenclature systems and the frequent use of alternative names as new interleukins were discovered by more than 1 group has led to a great deal of confusion and ambiguity. A nomenclature for lymphokines was proposed in 1979,^4^ and subsequently, a common interleukin nomenclature system was approved by the International Union of Immunological Societies and the World Health Organization Nomenclature Subcommittee.^5,6^ A new nomenclature system based on peptide sequence conservation, common structural elements, gene architecture, and publication date was suggested for IL-1 family genes.^7^ More recently, interleukins have been classified by genomic architecture and characteristic protein structural features into 4 major groups, with a number of unclassifiable outliers. ^8^ The most recent comprehensive classification of interleukins from IL-1 to IL-38 uses a combination of sequence homology, receptor chain similarities, and functional properties.^2^

## METHODS

### Provenance and review process

Information in Reactome is captured from published scientific literature by using postgraduate curators. Every new event undergoes peer review by domain experts and cites reference publications that contain the original experimental data used to determine the event details. Publications that report experiments conducted with human reagents are given preference because these are most likely to reflect *in vivo* human biology. If an event has only data obtained by using model organisms, it is clearly annotated as an inferred event. Details of the event as it occurs in the model organism are included in the database, and links to the experimental data are provided, allowing the user to satisfy himself or herself that results can be extrapolated reliably to the equivalent human event. All such inferred events are checked by the expert reviewer to ensure that the inference from model organism to human is valid. Reviewers are asked to check the accuracy of molecular details and to ensure that events represent the consensus of knowledge in the field and not findings that are controversial or recent unconfirmed reports.

### Annotation

Reactome extensively cross-references external databases, such as Ensembl, ^9^ Gene Ontology (GO),^10^ PubMed,^11^ Chemical Entities of Biological Interest (ChEBI),^12^ UniProt,^13^ Online Mendelian Inheritance in Man (OMIM),^14^ IntAct,^15^ and many others related to disease, genetic disorders, ontology, and literature, enriching the information that can be accessed.

### Hierarchical organization

**FIG 1:**
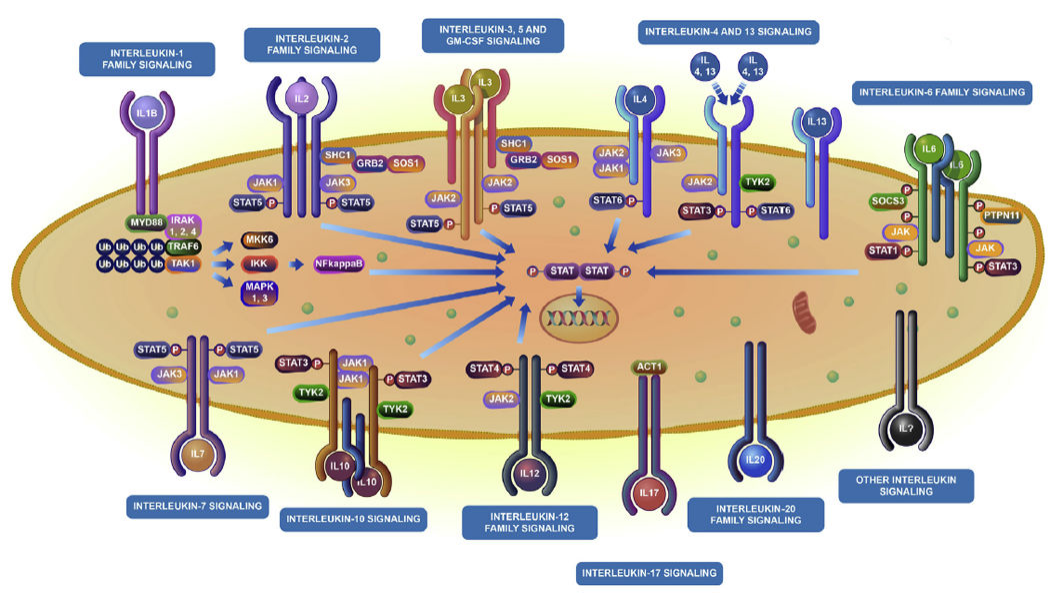
Enhanced high-level diagram *(EHLD)* for signaling by interleukins. EHLDs use familiar and consistent graphic elements that identify the regions of the graphic that are links to subpathways, which represent full molecular details. In this example interleukins are represented as *circles*, and interleukin receptors or families are represented as *pairs or trios of rods with cupped ends*. Further icons provide a minimal and generalized representation of signaling. The EHLD design incorporates labels as part of the selectable region for users who are not able to interpret the iconography. The figure has been copied under CC-BY with permission from Reactome. An interactive version with links to detailed subpathway representations is accessible at http://reactome.org/PathwayBrowser/#/R-HSA-449147.

**FIG 2:**
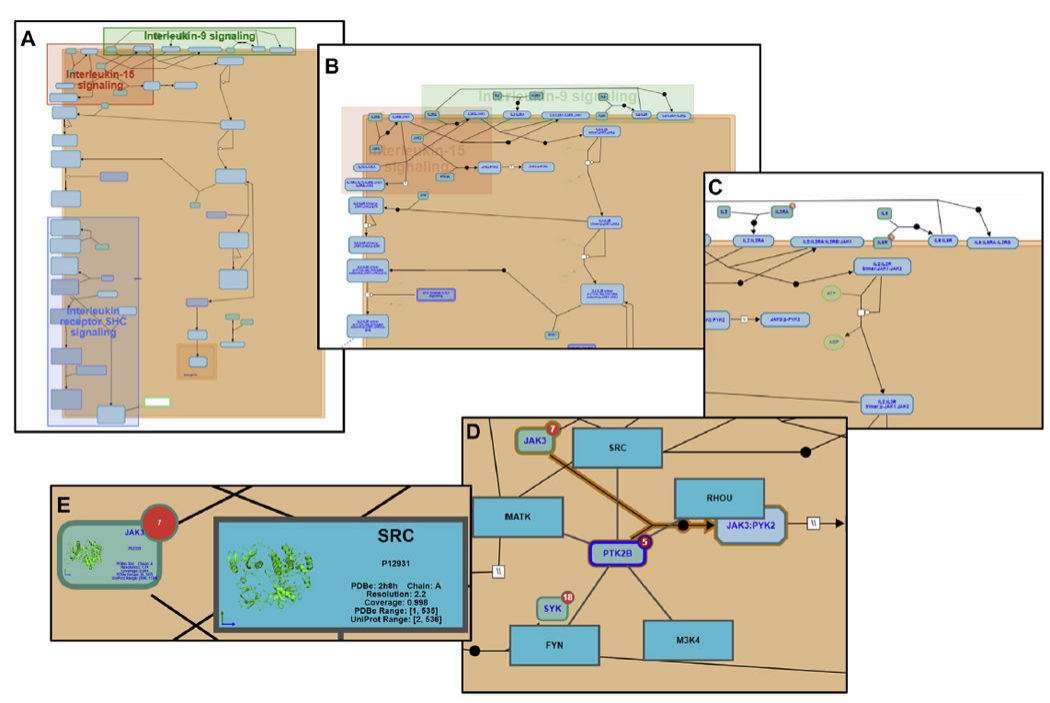
Pathway diagram for IL-2 family signaling showing the increase in detail level as zoom level increases. **A**, Lowest zoom level, with *colored boxes enclosing shaded regions* representing subpathway locations. **B**, Appearance of labels for molecular objects (eg, proteins and complexes). **C**, Fade-in appearance of trivial molecules and interactor labels *(red circles)*. **D**, Display of interactors in radial layout around the pathway molecule. **E**, Three-dimensional structure appearance.

Reactome is organized hierarchically. At the highest level are pathways representing broad biological function, such as the immune system. These typically group together several subpathways, such as the adaptive immune system, innate immune system, and cytokine signaling. This organization largely follows the Gene Ontology Biological Process hierarchy.^16^ Although higher level pathways aggregate pathways within the same broader biological process, at lower levels of the hierarchy, pathways represent the molecular details of the pathway as a series of molecular events. In both cases the data are stored in a formal structured data model and made available as interactive diagrams. ^17^ Higher-level pathways are represented by enhanced high-level diagrams that are intended to visually guide users to the specific subpathway topics that interest them (Fig 1). Lower-level pathways are represented as pathway diagrams, which use a standard graphic representation of the molecular events that constitute the pathway (Fig 2).

### Reactome analytic tools

The Reactome Web site has several tools that can be used to submit a user data set for analysis and visualization of pathway overrepresentation (enrichment) and representation of expression data viewed as an overlay on Reactome pathways. Protein-protein and protein-compound interactors can be visualized as potential extensions to Reactome’s curated pathways. Interactors are available for more than 10 source databases or can be submitted as a custom interactions data set. This provides a useful approach for identifying candidate receptor-binding partners or pathway-signaling extensions, potential tool compounds that could be used to modulate the pathway, or novel lead compounds. Using the analytic tools on theWeb site or through the analysis services, users can easily discover whether the set of proteins they have found to be associated with a particular treatment or disease state is clustered in a particular biological process. They can take advantage of disease-associated pathway visualizations to determine whether a mutation that blocks the function of a protein of interest would directly perturb a biological process. Interpretation of these analyses is facilitated on the Web site by means of dynamic navigation and detail-level adjustments between views, features that enable visualization of the connections between pathways and domains.^18^ Representation of data as pathway diagrams has been shown to support interpretation more efficiently than equivalent linguistic representations.^19^

A protein-naming convention developed for Reactome^20^ is used throughout the signaling by interleukins pathway to avoid the confusing plethora of alternative naming systems present in the historical literature. At the same time, the diverse names given various entities have been preserved as synonyms that are indexed by our search tools, so that this naming history is readily accessible.

## RESULTS

### Interleukins and their classification

Interleukins have been classified into families by using a variety of characteristics, including biological function, shared receptor components, sequence homology, structural motifs, and cellular origin. The classification used in Reactome is derived largely from Akdis et al^2^ and IUPHAR.^21^ Some of the most recent interleukins are not represented in the IUPHAR categorization (eg, IL-14, IL-37, and IL-38). Reactome includes some interleukin-related cytokines that are not represented by Akdis et al^2^ (eg, ciliary neurotrophic factor, leukemia inhibitory factor, and oncostatin M).

Interleukin signaling in Reactome is organized within the pathway signaling by interleukins. This pathway has subpathways that represent the molecular events of signaling of specific interleukins or interleukin families in an associated pathway diagram. Some such families contain several well-studied interleukins, each with an extensive signaling pathway. Family members are typically grouped together within the hierarchical organization and graphic views.

In a few cases the graphic representation of members of a family has been split into several pathway diagrams to allow complete representation of the molecular details in an uncluttered graphic. For example, IL-4 and IL-13 are typically considered part of the IL-2 family, but their signaling pathways are placed at the same level of Reactome’s pathway hierarchy and represented graphically as separate diagrams. This is consistent with Akdis et al,^2^ who represent IL-4 and IL-13 as a branch of the IL-2 family.

Other interleukins with well-studied and consequently extensive signaling pathways that are represented in a dedicated pathway diagram include IL-1, IL-2, IL-6, IL-7, IL-9, IL-10, and IL-17. Interleukins grouped by shared receptor subunits include IL-4 and IL-13, which share a receptor that includes the IL-4 receptor, and IL-3, IL-5, and GM-CSF, which use receptors that share IL-3RB (the common β chain). Other interleukins are grouped into recognized families, such as the IL-6 family, which typically includes IL-11, leukemia inhibitory factor, CT-1, oncostatin M, ciliary neurotrophic factor, and IL-31. Although in some classifications IL-27 is placed in the IL-6 family,^22^ we follow the more recent classification of Akdis et al,^2^ which places IL-27 in the IL-12 family.

### Programmatic access

The formal data model embodied in Reactome’s representation of pathways makes its content consistent and computationally accessible. This content is available programmatically through a REST-based content service and as a Neo4J graph database. Reactome provides visualization widgets that can be embedded into Web pages, making Reactome content easily reusable in third-party Web sites, benefiting the systems biology and modeling communities. Reactome offers a pathway analysis service for enrichment and expression analysis.^23^ An online description of all the features of the Reactome Web site is available at: http://www.reactome.org/userguide/Usersguide.html.

### Training and help

Reactome training is available in the form of a webinar and self-paced online courses through the EBI Train Online Web site (https://www.ebi.ac.uk/training/online/course-list?field_course_ subject_area_tid%5B%5D519).

Help is available through the “Contact Us” link on the Reactome homepage or by e-mailing help@reactome.org.

## DISCUSSION

### Interleukins in Reactome

Reactome describes the signaling pathways of specific interleukins as a series of molecular events linked to each other by preceding-following annotations. The event-level relationship shows a biological connection of individual events within a selected pathway and also of distinct pathways in the hierarchy. For example, the Reactome annotations of IL-1 family members include gene transcription, caspase-1 (CASP1)– or CASP8- mediated processing of precursors, secretion of the active protein, receptor binding, and downstream signaling events. The event of CASP1-mediated IL-1 processing belongs to the IL-1 family signaling pathway and is linked to the downstream events as the preceding event. At the same time, this cleavage reaction is followed by CASP1 activation, which is in turn induced on inflammasome formation. Thus the events of the CASP1 activation bridge IL-1 family signaling pathway to the upstream intracellular signaling processes described in the pathway of inflammasomes.

Within the Reactome Pathway Browser, the signaling by interleukins pathway uses an illustrative graphic representation^17^ that contains glyphs representing interleukin subpathways (Fig 1). When selected, they link to more detailed pathway diagrams of individual pathways or pathway groups that represent the molecular events involved in signaling by the selected interleukins (Fig 2).

Selecting any of the clickable regions links the user to details of the corresponding subpathway. Specifically, the display updates to show a detailed pathway diagram (Fig 2). Pathway diagrams represent pathways as connected detailed molecular events based on an iconography known as Systems Biology Graphical Notation, a recognized standard for the representation of pathway information. ^24^ A full description of the representation used in pathway diagrams is available in the “Reactome user guide” (http://wiki.reactome.org/index.php/Usersguide). A diagram key is available within the Reactome Pathway Browser, as revealed by selecting the button with a compass icon (Fig 1, top right corner). In brief, individual protein molecules are represented as green boxes, complexes as blue boxes with the corners removed, sets of interchangeable molecules with a common function as blue boxes with a double boundary, and small molecules as green circles or ovals. Cellular compartments are represented as pink/brown boxes surrounded by a darker double boundary line that represents the enclosing membrane, such as the cytosol boundary represents the plasma membrane. The white diagram background represents the extracellular space. Events are represented in diagrams through a central node, which is connected by simple lines to boxes representing inputs to the event and by arrowed lines to boxes representing outputs of the event. The output of an event might be an input to another event, thereby representing 2 connected events within the same pathway process.

The level of detail visible within a pathway diagram adjusts depending on the size of the presentation area and the level of zoom. At the lowest level of zoom, any subpathways within the pathway diagram are indicated by enclosing shaded boxes (Fig 2, *A*). As zoom level increases, these shaded boxes fade from view, whereas the labels for boxes representing molecules fade into view (Fig 2, *B*). As zooming continues, the glyphs for trivial small molecules fade into view, as do the red circular icons on boxes that indicate the availability of protein-protein interactors (Fig 2, *C*). In Fig 2, *D*, the interactors for PTK2B are revealed, appearing as a radial layout or green-blue boxes around it. At the highest level of zoom, 3-dimensional structures and selected details appear inside the boxes representing proteins and protein interactors, if available from PDBe.^25^ Boxes for small molecules display chemical structures from ChEBI.^12^ All the molecular objects displayed within pathway diagrams can be selected. This action updates the details panel section of the pathway browser (not shown). For events, the details include a description of the selected object or event and literature references containing experiments that provide evidence supporting the molecular details of the event.

The addition to Reactome of a complete set of the known human interleukins, their receptors, and established signaling pathways linked to annotations of relevant aspects of immune function provides a significant computationally accessible resource of information about this important family. Reactome content is dynamic and intended to expand and undergo revision as new discoveries are published and become accepted. A key ongoing aim is to increase coverage of gene expression changes induced by interleukin signaling as these data become confirmed and established.

## Key messages

- Reactome is a free online database of human biological pathways.
- Pathways are peer reviewed and aim to represent the established consensus.
- The signaling processes mediated by IL-1 to IL-38, their receptors, and accessory proteins are integrated into the major topic of the immune system.
